# The Treatment of Eczema with Picric Acid

**Published:** 1897-11

**Authors:** 


### The Treatment of Eczema with Picric Acid.

In an article on this subject in the *Nouveau Montpellier medical* for September, M. A. Brousse remarks that the kerato-plastic property of picric acid, which has been successfully used in burns, seems to indicate that its employment is proper in the treatment of eczema, certain forms of which present great analogies to superficial burns. In 1889, he says, Cerasi employed this drug in seven cases of eczema with excellent results. Dr. McLennam, of Glasgow, was also very successful in the treatment of acute eczema and eczema of the face with this drug, which he used in a saturated solution. The author himself has obtained rapid recovery in several cases in which he has employed this treatment, the histories of which are given in detail. In cases of lichenoid eczema with a thick epidermis the acid was useless, but in acute oozing eczema, accompanied by œdema of the skin, it was very useful. Under its influence in one case recovery was obtained in two weeks; in another case, in ten days.

Among the advantages of this treatment are the immediate relief produced by the application of the picric-acid solution and the disappearance of the pain, heat, and itching; the rapidity with which œdematous tumefaction is effaced; and the absolute painlessness of the dressing, even when it is applied to the bare surface of the derma. According to the opinion of the most competent observers, the extensive application of this drug does not give rise to any symptoms of poisoning. Not only is it useful in acute eczema, but it is also useful in the acute attacks of chronic eczema which are so frequent in arthritics, particularly if they are accompanied by oozing and ulceration of the skin; it is equally useful in the seborrhoic eczema of infancy. The author states that the results obtained by him with this treatment absolutely confirm those indicated in the publications of

Dr. McLennan, M. Gaucher, and M. Leredde. M. Brousse therefore concludes that this treatment is indicated as follows: 1. In acute eczema. 2. In the acute attacks of chronic eczema, particularly if there is a tendency to oozing and ulceration of the skin. 3. In the seborrhoic eczema (impetiginous) of infancy. This treatment, he says, is contraindicated in chronic eczema and generally in all those forms of eczema which are accompanied by a thickening of the epidermis (lichenoid eczema). Nevertheless, it has the advantage, even in these cases, of allaying the itching.—*N. Y. Medical Journal.*

